# Effect of low-level ultrasound treatment on the production of L-leucine by *Corynebacterium glutamicum* in fed-batch culture

**DOI:** 10.1080/21655979.2021.1906028

**Published:** 2021-03-29

**Authors:** Yufu Zhang, Zhichao Chen, Pengjie Sun, Qingyang Xu, Ning Chen

**Affiliations:** a National and Local United Engineering Lab of Metabolic Control Fermentation Technology, Tianjin University of Science & Technology, Tianjin, PR China; b College of Biotechnology, Tianjin University of Science & Technology, Tianjin, PR China

**Keywords:** Ultrasound, l-leucine, fermentation, *corynebacterium glutamicum*, response surface methodology, fed-batch culture

## Abstract

Various process intensification methods were proposed to improve the yield, quality, and safety of fermented products. Here, we report the enhancement of L-leucine production by *Corynebacterium glutamicum* CP using ultrasound-assisted fed-batch fermentation. Response surface methodology was employed to optimize the sonication conditions. At an ultrasonic power density of 94 W/L, frequency of 25 kHz, interval of 31 min, and duration of 37 s, *C. glutamicum* CP produced 52.89 g/L of L-leucine in 44 h, representing a 21.6% increase compared with the control. The production performance of L-leucine was also improved under ultrasonic treatment. Moreover, the effects of ultrasound treatment on the fermentation performance of L-leucine were studied in terms of cell morphology, cell membrane permeability, and enzyme activity. The results indicate that ultrasonication is an efficient method for the intensification of L-leucine production by *C. glutamicum* CP.

## Graphical abstract

**Figure:**
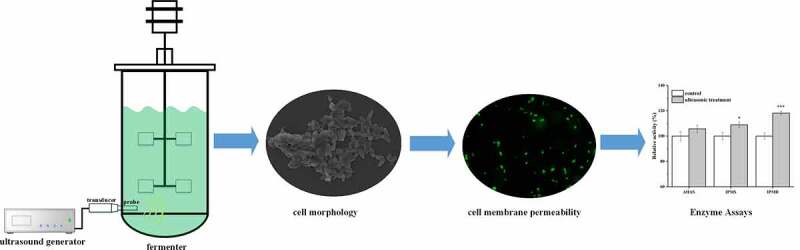

## Introduction

L-leucine, is one of eight essential amino acids that cannot be synthesized by mammals, with important roles in physiological functions and metabolism [1–3]. L-leucine is naturally found in food, and is also used in the pharmaceutical industry, cosmetics, as a precursor for antibiotics and herbicides, or as a food additive [4]. Generally, L-leucine can be produced by protein hydrolysis and extraction, chemical synthesis, enzymatic methods, and microbial fermentation. With the rising market demand for L-leucine, fermentation methods have attracted increasing attention due to their economic and environmental advantages.

The most common bacteria used for L-leucine production via fermentation are *Corynebacterium glutamicum* and *Escherichia coli* [5,6]. Most early L-leucine production strains were the product of random mutagenesis and screening, but this approach introduces unclear genetic alterations, which may cause unwanted effects such as, growth retardation, increased by-product formation, and genetic instability [7]. Consequently, recent studies on L-leucine production relied on targeted genetic manipulation to avoid this problem. Genetically defined strains with high productivity can be created by improving the supply of precursors, releasing feedback inhibition, blocking competing pathways, and overexpressing genes related to the target amino acid synthesis pathways [4,8,9]. There are a number of reports on the metabolic engineering of microbial cells for improved L-leucine production [10–12].

With *C. glutamicum* ATCC13032 taken as the chassis strain, an efficient L-leucine production strain, MV-LeuF2, can accumulate L-leucine to levels exceeding 24 g/L under fed-batch culture conditions [10]. Although great strides have been made in the development of L-leucine-producing strains, process control and technology are essential to maximize the production performance of the strains and achieve industrially relevant yields. As a non-thermal physical processing method, ultrasound treatment has been widely investigated for applications in the food industry, especially in fermentation engineering [13,14]. Ultrasound technology can be used as a process intensification method to achieve safer, cleaner, and more energy-efficient production modalities. However, while low-level ultrasound can stimulate the growth of microorganisms depending on the intensity and frequency of the applied ultrasound, inappropriately calibrated ultrasound treatment can easily be lethal [15]. Ultrasound can alter the metabolic activity of microbial cells, thus accelerating proliferation, increasing enzyme production and metabolite titers, as well as increasing the membrane permeability [16,17]. Similarly, low-intensity ultrasound has been reported to increase the membrane permeability and hydrophobicity of biofilm-forming microorganisms, exerting positive effects on biofilm formation by increasing the delivery of oxygen and nutrients to the deeper layers [18,19].

Beneficial effects of low-level ultrasound on fermentation have been observed in several microorganisms [20]. In general, Gram-positive bacteria are more resistant to ultrasound than Gram-negative bacteria due to their characteristic cell walls, wall thickness and the cross-linking of peptidoglycans that makes them more robust [21]. For instance, the acetone-butanol-ethanol (ABE) yield of *Clostridium acetobutylicum* MTCC 11274 in an ultrasound-assisted fermentation process reached 0.288 g/g raw biomass after 92 h, compared with 0.168 g/g raw biomass after 120 h using only mechanical agitation [22]. Similarly, ultrasonic treatment was found to increase the production rate of GSH by *S. cerevisiae* [23]. Furthermore, ultrasonic treatment was found to increase the membrane permeability and substrate utilization of mixotrophic microalgae without significantly reducing their viability, resulting in a significant increase of biomass and lipid accumulation [24]. Due to these promising results and application prospects, ultrasound technology has attracted increasing attention in fermentation engineering.

In this study, we used low-intensity sonication to intensify the L-leucine fermentation process. We investigated the effects of different ultrasound power densities, frequencies, intervals, and durations on the growth and L-leucine production of *C. glutamicum* CP, finally identifying the optimal combination using response surface methodology (RSM). We also analyzed the effects of ultrasound on cell morphology, cell membrane permeability, and activity of key enzymes to understand the mechanism of fermentation enhancement by ultrasound treatment.

## Materials and methods

### Strain and culture media

The leucine-producing strain *Corynebacterium glutamicum* CP was engineered through multiple rounds of random mutagenesis and screening [25]. The complete genome sequence of *C. glutamicum* CP was reported by Gui et al. [26].

The medium used for seed culture contained (per liter): 40 g glucose, 40 g corn steep liquor (CSL), 2 g KH_2_PO_4_ · 12H_2_O, 2 g MgSO_4_ · 7H_2_O, 5 mg FeSO_4_ · 7H_2_O, 0.2 g L-methionine, 0.3 g L-isoleucine, 0.5 mg biotin, and 0.2 mg thiamine.

The fermentation medium consisted of (per liter): 100 g glucose, 20 g CSL, 2 g KH_2_PO_4_ · 12H_2_O, 3 g MgSO_4_ · 7H_2_O, 30 mg MnSO_4_·H_2_O, 30 mg FeSO_4_ · 7H_2_O, 0.2 g L-methionine, 0.3 g L-isoleucine, 2 g L-glutamic acid, 0.3 mg biotin, and 0.3 mg thiamine. All media were adjusted to pH 7.2 with NaOH. All reagents were purchased from Sinopharm Chemical Reagent (China).

### Fed-batch cultivation

Fed-batch cultivations were performed in a 30-L fermenter (Baoxing Biological Equipment Engineering Co., LTD, Shanghai, China). Bacterial growth was monitored by measuring the optical density at 600 nm (OD_600_). When the cells reached the mid-exponential phase (OD_600_ = 15–20), the seed culture was transferred into the bioreactors with the indicated inoculum size. Culture temperature was maintained at 32°C and pH was maintained at 7.0–7.2 by automated addition of ammonium hydroxide (25%, v/v). the dissolved oxygen saturation (DO) was kept at 20–30% by adjustment of the agitation and aeration rates. Antifoam 204 (Sigma-Aldrich, China) was added to the bioreactor to prevent foam formation when necessary. When the concentration of glucose in the medium fell below 10 g/L, feed solution (80% glucose, w/v) was added to maintain a residual glucose concentration of approximately 5 g/L in the feed phase.

### Determination of the optimal ultrasonic treatment scheme

The fermentation system consisted of a fermenter and multi-frequency power ultrasonic equipment (Handan Haituo Machinery Technology Co. LTD, Hebei, China). The ultrasonic system included an ultrasound generator, transducer, and a probe attached to the bottom of the fermenter (Fig. S1). The single-factor experiment included two parts: exploring the effect of ultrasound treatment in different growth phases, and the effects of ultrasonic power density (50–250 W/L), frequency (15–40 kHz), interval (10–50 min), and duration (20–60 s) on the growth of *C. glutamicum* CP.

### RSM analysis of the optimal ultrasonic treatment scheme for the production of L-leucine

RSM has been used in several ultrasound studies [27–29]. For instance, RSM coupled with Box–Behnken design was chosen to identify relations between the responses (*in vitro* angiotensin-I-converting enzyme inhibitory activity, peptide content, and biomass of *B. subtilis*) and some ultrasonic treatment parameters [30]. The production of L-leucine was performed in a growth-coupled process, so the optimum ultrasonic conditions for L-leucine production were based on the single-factor experiments described above. The influence of ultrasonic power density, frequency, interval, and duration on L-leucine production was investigated using RSM. Design Expert statistical software (version 8.0.6, Stat-Ease, Inc., USA) was used for the experimental design and statistical analysis. To evaluate the influence of the parameters and their interactions on the response surface, a three-level, four-factor Box–Behnken design was employed. The experimental variables were investigated at three levels (−1, 0, +1; Table S1). The titer of L-leucine produced by *C. glutamicum* CP was selected as the response vector, Y (g/L). The response variables were fitted to the second-order polynomial model equation (1), which describes the relationship between the responses and independent variables.

Eq. (1): Y = β_0_ + Aβ_1_ + Bβ_2_ + Cβ_3_ + Dβ_4_ + ABβ_5_ + ACβ_6_ + ADβ_7_ + BCβ_8_ + BDβ_9_ + CDβ_10_ + A^2^β_11_ + B^2^β_12_ + C^2^β_13_ + D^2^β_14_

where Y is the response (L-leucine titer), A is the ultrasound power density, B is the ultrasound frequency, C is the ultrasonication interval, D is the duration of ultrasonication, β_0_ is a constant, β_1_–β_4_ are linear coefficients, β_5_–β_10_ are interaction coefficients between the factors, and β_11_–β_14_ are quadratic coefficients.

### Analytical methods

Samples comprising 5 mL of the fermentation broth were taken every 4 h for analysis. Cell growth was determined by detecting changes in dry cell weight (DCW). The cells were collected by centrifugation (8000 × *g*, 10 min, 4°C) and then washed with distilled water, and dried at 105°C until achieving a constant weight.

The SBA-40E immobilized enzyme biosensor (Biology Institute of Shandong Academy of Sciences, Jinan, China) was employed to measure the concentration of residual glucose in the culture supernatant. The L-leucine concentration was measured by high-performance liquid chromatography (HPLC) using an LC20AT system (Shimadzu, Kyoto, Japan) equipped with an Agilent ZORBAX Eclipse AA column (4.6 × 150 mm, 5 µm; Agilent Technologies, Palo Alto, CA, USA) and a UV detector (360 nm). 50% acetonitrile and 50 mM sodium acetate were used as the mobile phase at a flow rate of 1 mL/min [25]. All samples were measured in triplicate.

### Preparation of the crude enzyme solution and determination of enzyme activity

To prepare the crude enzyme solution for measuring the enzyme activities of acetohydroxyacid synthase (AHAS), isopropylmalate synthase (IPMS), and 3-isopropylmalate dehydratase (IPMD), the cells were collected by centrifugation (8000 × *g*, 10 min, 4°C) and washed with 50 mM Tris-HCl (pH 7.4). Then, the resuspended cells were lysed by sonication, and the resulting crude lysate centrifuged (40,000 × *g*, 30 min, 4°C) to remove cell debris. The cleared supernatant constituted the crude enzyme solution. The protein concentration was determined using a BCA Protein Assay Kit (Solarbio, Beijing, China). The protein concentrations and enzyme activities were measured in triplicate.

AHAS enzyme activity was determined according to a published method [31]. One unit of enzyme activity was defined as the amount of enzyme required to produce 1 µmol of acetolactate per minute under the assay conditions.

IPMS enzyme activity was determined by detecting coenzyme A formation using Ellmann’s reagent [10]. One unit of enzyme activity was defined as the amount of enzyme that converts 1 µmol of α-isopropylmalate per min under the assay conditions.

IPMD enzyme activity was determined by detecting the production of the reaction intermediate α-isopropyl maleate [10]. One unit of enzyme activity was defined as the amount of enzyme that converts 1 µmol of α-isopropyl maleate per min under the assay conditions.

### *Scanning electron microscopy (SEM) analysis of* C. glutamicum *CP*

Samples of fermentations with or without ultrasonic treatment were centrifuged at 8000 × *g* for 10 min, the cell pellet was washed with a 0.85% NaCl solution three times, and the cells fixed in 4% glutaraldehyde solution for 3 h. The fixed cells were rinsed with 0.85% NaCl solution three times and further dehydrated using 50%, 70%, 90%, 95%, and 100% ethanol. Finally, the samples were dried at 37°C for 3 h, placed on sample stubs with conductive carbon tape, and sputter-coated with gold before microscopy using a JSM-5800LV SEM (JEOL, Japan) with a 10 kV beam.

### Measurement of cell membrane permeability

The LIVE/DEAD® BacLight^TM^ Bacterial Viability Kit L7012 (Thermo Fisher, USA) was used to assess membrane permeability. The kit consisted of SYTO 9 dye and propidium iodide (PI), which both stain nucleic acids. SYTO 9 stains live cells with intact membranes, and those with damaged membranes in green, while PI penetrates only cells with damaged membranes, causing a reduction in the SYTO 9 fluorescence when both dyes are present. Prior to analysis, cells from the stationary phase were washed three times with 0.85% NaCl and resuspended in 0.85% NaCl solution. Equal volumes of dye mixture were added to the bacterial suspension, mixed thoroughly and incubated at room temperature in the dark for 15 min. Stained cells were quantified using a BX53 fluorescence microscope (Olympus, Japan).

### Statistical analysis

The mean responses were fitted to a second-order polynomial using Design Expert 8.0.6 software to obtain regression equations, which were validated by calculating the coefficient of multiple determinations (R^2^). The statistical significance of the model was assessed using analysis of variance (ANOVA). All the experiments were performed in triplicate and the results were analyzed using SPSS software (IBM Corp., USA). The normality of data was tested using the Shapiro–Wilk test, and homogeneity of variances using Levene’s test. The statistical significance of differences was calculated using Duncan’s post hoc test, with P < 0.05 as the threshold.

## Results and discussion

Ultrasound is a versatile technology that can be used in conjunction with the fermentation process to increase the process efficiency and production rate by improving cell permeability and enzyme activity. We optimized the ultrasonic parameters, which significantly increased the biomass and the production of L-leucine. Furthermore, we investigated the differences in cell morphology, cell membrane permeability, and enzyme activity with ultrasound treatment.

### *Effect of ultrasonic treatment in different growth phases on the biomass of* C. glutamicum *CP*

**Figure 1.**
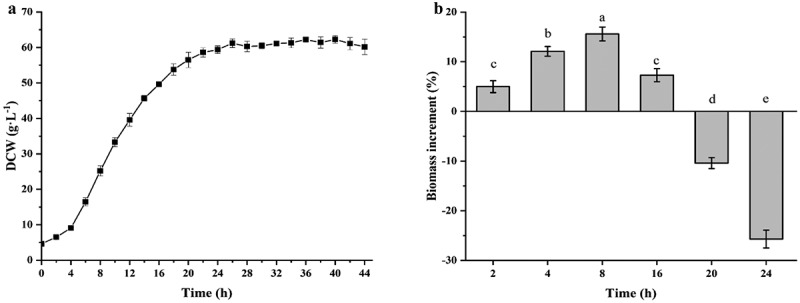
Effect of ultrasound treatment on the growth of *C. glutamicum* CP in different phases. (a): The growth curve of *C. glutamicum* CP; (b): biomass increase of *C. glutamicum* CP treated with ultrasound at different incubation times. Different letters indicate significant differences (P < 0.05)

The growth curve of *C. glutamicum* CP is shown in Figure 1a as baseline data for the identification of different growth phase. The cells were in the lag phase for the first 4 h, in the exponential phase from 4 h to 20 h, and entered the stationary phase after 20 h of culture. *C. glutamicum* CP was subjected to ultrasonic treatment at 2, 4, 8, 16, 20, and 24 h. The effects of ultrasound treatment on the different growth stages of *C. glutamicum* CP are shown in Figure 1b. At the beginning of the lag phase and exponential phase, there was a significant increase in biomass, and at 8 h, the growth of ultrasonically treated *C. glutamicum* CP increased by 15.6% compared to the control. However, ultrasound treatment in the late exponential and stationary phase resulted in a significant decrease of biomass. Ultrasound has a dual effect on microbes; a lethal effect or growth stimulation. The positive impacts on the cells growth are that increase the activity of enzymes and the mass transfer rate of the nutriment, while negative aspects include shear forces and free radicals produced by ultrasound [32]. Different growth stages of cells have different tolerance to ultrasound. As a result, ultrasonic treatment in the exponential phase at 8 h was selected for following experiments.

### *Effect of ultrasound parameters on the growth of* C. glutamicum *CP*

**Figure 2.**
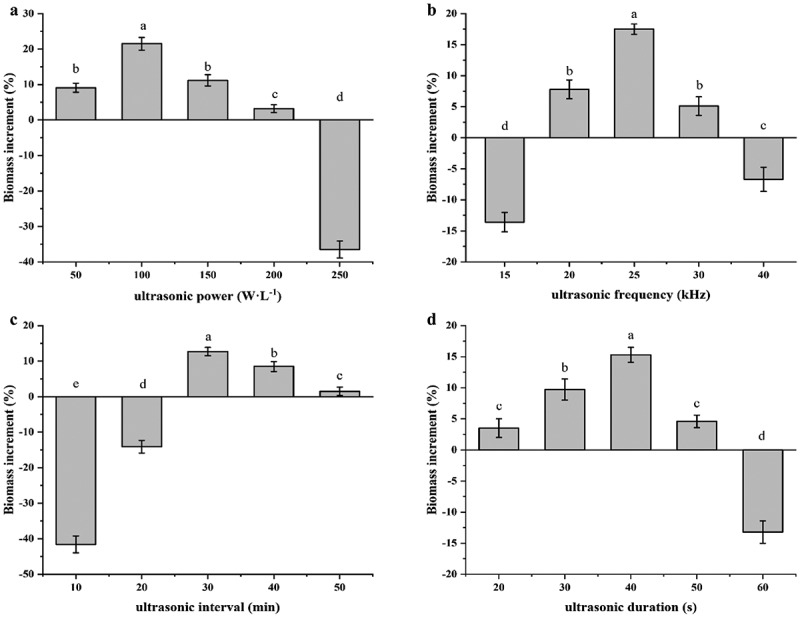
Effects of ultrasound treatment parameters on the growth of *C. glutamicum* CP. Biomass increase of *C. glutamicum* CP treated with the ultrasound at different power densities (a), frequencies (b), intervals (c), and durations (d). Letters indicate significant differences (P < 0.05)

Different powers and frequencies ultrasound generate either stable or transient liquid cavitation in liquid medium. Transient cavitation occurs at low frequencies of 20–100 kHz, while high frequencies over 200 kHz induce stable cavitation, resulting in thousands of oscillation cycles between high and low acoustic pressure [20,33]. To improve the biomass accumulation, single-factor experiments were carried out and the effects of different ultrasonication parameters on the growth of *C. glutamicum* CP were investigated, as shown in Figure 2. Various ultrasonic power densities (50, 100, 150, 200, and 250 W/L) were investigated (Figure 2a). All tested power densities enhanced the biomass to some degree, except for the highest setting of 250 W/L. At ultrasonic power densities of 50 and 150 W/L, there was no significant difference in biomass accumulation. The biomass reached a maximum with an increase of 21.5% over the control under an ultrasonic power density of 100 W/L. This indicates that lower ultrasonic power densities can stimulate the growth of *C. glutamicum* CP whereas over-stimulation with intensified ultrasound might result in cell damage. Thus, an ultrasonic power density of 100 W/L was used in the following experiments. Both too high and too low ultrasound frequencies had negative effects on the cell growth. Under ultrasonic stimulation at a frequency of 25 kHz, the biomass reached a maximum that was 17.5% higher than without ultrasonic treatment. Considering the high biomass, 25 kHz was the optimal frequency for ultrasound treatment in this study.

In addition to the power density and frequency, it is crucial to select an appropriate time of ultrasonic treatment, because serial sonication can easily cause irreversible damage to cells or even instruments [34]. Several studies reported the influence of pulsed ultrasonic models on the growth of microorganisms. It was found that pulsing with an on time of 100 s and off time 10 s at 28 kHz and 100 W/L for 0.5 h led to the highest increase in the peptide content and viable cell count [27]. Similarly, Ren et al. optimized the ultrasonic interval to improve the biomass and lipid accumulation of mixotrophic microalgae [24]. In this study, the ultrasonic interval was also found to have a great influence on the growth of *C. glutamicum* CP. However, the biomass decreased significantly with shorter ultrasonic intervals. A maximal biomass increase of 12.7% was obtained with an ultrasonic interval of 30 min (Figure 2c). Furthermore, the increase of *C. glutamicum* CP biomass with ultrasound treatment reached the maximum with ultrasound treatment for 40 s (Figure 2d).

### Modeling the effect of ultrasound treatment L-leucine production

The modeling results for L-leucine production are shown in Table S2. The R^2^ value, P value, and F value was calculated to evaluate the mutual interactions between the dependent and independent variables (Table S3). The data were in good agreement with a second-order polynomial model (Eq. (1)), with an R^2^ value of 0.9747, which suggested that the model could be used to optimize the ultrasonic treatment conditions for improved L-leucine fermentation. The model was highly significant (P < 0.01), and the lack of fit indicated a good correlation with the model data (P < 0.05). Furthermore, the rather low coefficient of variance (CV< 10%), indicated low dispersion degree of the data in the model. This further supports the good fit of the model, and thus, indicates good reproducibility. The second-order polynomial Eq. (2) describes the relationship between ultrasonic power density (A), frequency (B), interval (C), and duration (D). E represents scientific notation (E).

Y = −526.26592 + 0.80385A + 27.23841B + 4.79884 C + 5.99755D – 0.012437AB + 0.012781AC – 1.94466E – 003AD – 0.070948BC – 0.036414BD – 9.12218E – 003 CD – 4.37387E – 003A^2 –^ 0.43179B^2 –^ 0.060933C^2 –^ 0.060604D^2^ (2)

### Optimization of ultrasonic treatment conditions by RSM analysis

**Figure 3.**
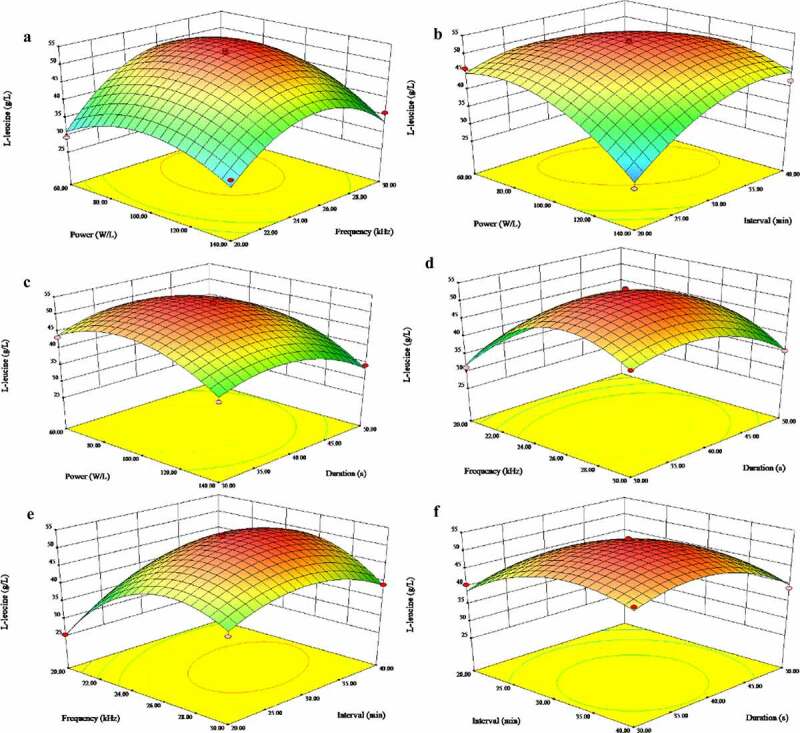
Response surface plot for interactions between four independent variables affecting L-leucine production. The titer of L-leucine was observed as a response variable for the interaction of two independent variables. The other parameters were at central points. Two variables were plotted against each other in each panel

In this study, the four independent variables showed a linear effect on the production of L-leucine under ultrasonic treatment, according to the regression analysis. As shown in Table S3, L-leucine was significantly affected by ultrasonic power density, frequency, interval, and duration (P < 0.01 in all cases). The interactions of power density *versus* frequency, power density *versus* interval, and frequency *versus* interval were statistically significant (P < 0.05), while the interactions of power density *versus* duration, frequency *versus* duration, and interval *versus* duration were not significant (P > 0.05). The L-leucine titers reached their highest level close to the midpoint of the response plot. The influence of these four variables on L-leucine production was further analyzed using three-dimensional plots (graphical representations of the regression model, Figure 3). The shape of the response surface curves indicated a moderate interaction between the assigned variables. The interactions between any two factors can be understood intuitively using response surface plots, and it is also convenient to locate their optimal levels. The L-leucine titer was observed as a response variable in the interaction between the ultrasonic power density and frequency, while the two other parameters were at central values. Furthermore, the L-leucine titer at an optimal value of ultrasonic power density and frequency could be calculated. L-leucine production was enhanced at the ultrasonic power density and frequency of intermediate levels (Figure 3a). The same course of the remaining factors (Figure 3b–F) indicated that the optimal value of each parameter could be obtained.

**Figure 4.**
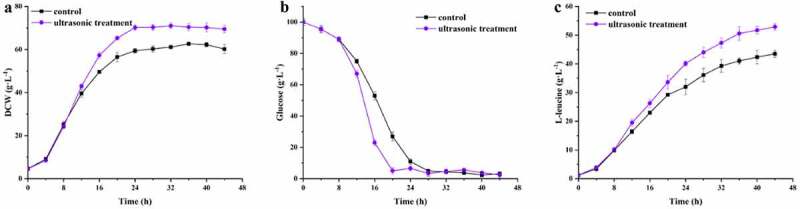
Time profiles of biomass (a), glucose (b), and L-leucine (c) concentrations in fed-batch fermentation of *C. glutamicum* CP with and without ultrasonic treatment. All fermentation experiments were performed in three independent replicates (n = 3)

The optimal conditions for L-leucine production were obtained by applying the prediction profiler model with the following data: ultrasonic power density of 94.00 W/L, frequency of 25.47 kHz, interval of 31.13 min, and duration of 37.04 s. Under the optimal ultrasonic treatment conditions, the maximal predicted L-leucine titer was 53.36 g/L. Validation experiments were performed in triplicate. The ultrasound treatment under the optimized conditions started at 8 h of fed-batch fermentation. The experimental conditions encompassed an ultrasonic power density of 94.00 W/L, ultrasonic frequency of 25 kHz, ultrasonication interval of 31 min, and duration of 37 s. The biomass and L-leucine concentrations in the ultrasonic experiments were significantly higher than in the control (Figure 4). The L-leucine titer was 52.89 g/L, which was about 21% higher than in the control (43.5 g/L), and was similar to its predicted value (53.36 g/L) according to the equation. The errors between the predicted and experimental values were < 2%. Thus, the regression models obtained by RSM could predict L-leucine production by any combination of independent sonication variables.

The yield of L-leucine from glucose under ultrasound treatment was 0.30 mol/mol, and the productivity was 1.2 g/L/h. These values represent 8.2% and 14.2% increases over the control, respectively. Therefore, low-level ultrasound treatment can improve the fermentation profile and productivity of L-leucine by increasing the viability, enzyme activity, and membrane permeability of microbial cells. Therefore, ultrasound can be applied to L-leucine production if optimal ultrasonication parameters are carefully determined before applying sonication.

### *Effects of ultrasonic treatment on the cell morphology of* C. glutamicum *CP*

**Figure 5.**
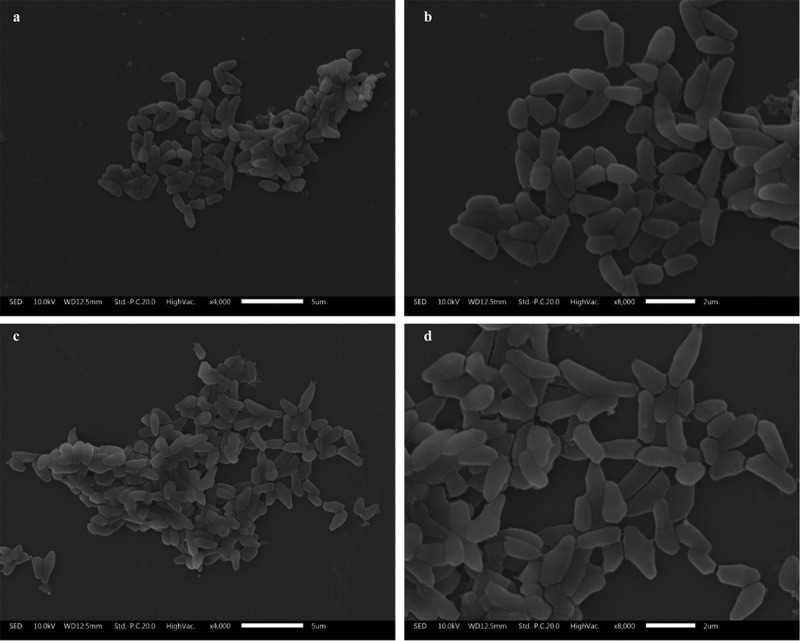
Electron microscopic observation of the cell morphology of *C. glutamicum* CP with/without ultrasonic treatment. (a–b): control cells; (c–d): ultrasound treated cells

SEM is widely used to assess the surface characteristics, morphology, and ultrastructure of microorganisms. We examined the effects of ultrasonic treatment on the cell morphology by SEM. The *C. glutamicum* CP cells showed a typical asymmetric rod shape, and V-shaped cell pairs were observed frequently (Figure 5). However, there were more irregular, elongated, and swollen cells in the ultrasonicated culture than in the control. After low-level ultrasonic treatment, *C. glutamicum* CP can survive and maintain the integrity of its cell structure. *C. glutamicum* CP, a Gram-positive bacterium, was more resistant to low-level ultrasound due to its cell wall characteristics. Though the positive effect of low intensity ultrasound was reported [20], ultrasound had a certain damage effect on cell wall. Fortunately, ultrasound was not continuous, which enabled cells to repair these damages in the ultrasonic interval, maintained a complete cell morphology, and avoided death. SEM images of untreated and ultrasonically treated cultures can provide insights into the growth and metabolism of *C. glutamicum* CP.

### Effect of ultrasonic treatment on cell permeability

**Figure 6.**
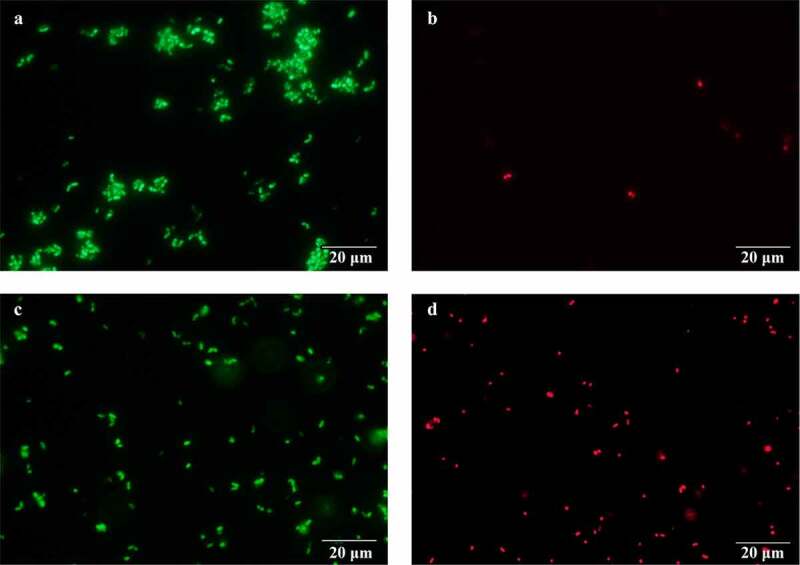
Fluorescence microscopy of *C. glutamicum* CP cells with/without ultrasound treatment. The cells exhibiting green fluorescence have an intact membrane, while cells with a permeabilized membrane exhibit red fluorescence. (a–b): control cells; (c–d): ultrasound treated cells

The cell membrane separates the cytoplasm from the environment and plays an important role in reproduction, energy transfer, and metabolism [35]. To determine whether ultrasonic treatment affects the integrity of the *C. glutamicum* CP cell membrane, a viability stain was used in conjunction with fluorescence microscopy. When double-stained with PI and SYTO 9, cells with intact membranes show green fluorescence, while damaged membranes allow the influx of PI, leading to red fluorescence [36]. As shown in Figure 6, the number of cells that emitted a green fluorescence was reduced under ultrasound compared with the untreated controls, while the number of cells emitting red fluorescence increased. This indicated that the *C. glutamicum* CP cell membrane was damaged to some extent under ultrasound treatment, which may have improved L-leucine production by enhancing nutrient transport. Increased membrane permeability also leads to intracellular efflux of L-leucine, which mitigates transcriptional attenuation of key genes and feedback inhibition of key enzymes in the L-leucine biosynthesis pathway. This is one of the reasons for the increased production of L-leucine by ultrasound. Additionally, stable microbubble oscillations can also lead to the production of H_2_O_2_ and other reactive oxygen species, which cause lipid and membrane proteins peroxidation. It was suggested that lipid and membrane proteins peroxidation might be one of the reasons increasing membrane permeability. Another possible reason for the enhancement of membrane permeability is acoustic cavitation, which is directly responsible for the mechanical effects of ultrasound treatment [37]. Ultrasound can increase the permeability of cell membrane both physically and chemically.

### Effects of ultrasonic treatment on the activity of key enzymes

**Figure 7.**
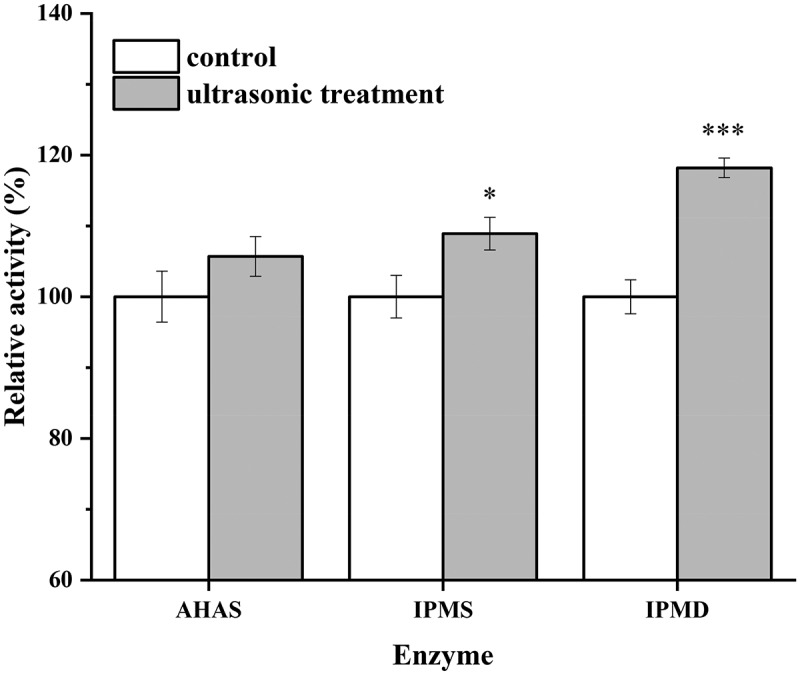
Relative activity of AHAS, IPMS, and IPMD in cells from the stationary phase with and without ultrasound treatment. The enzyme activities in the control were set 100%. The data represent the means ± standard deviations from three independent experiments. *P ≤ 0.05, **P ≤ 0.01, ***P ≤ 0.001。

AHAS, IPMS, and IPMD are key enzymes in the bacterial L-leucine biosynthesis pathway. In *C. glutamicum*, only one AHAS is encoded by the *ilvBN* gene. The *leuA* gene encodes IPMS, and the *leuCD* genes encode IPMD [38]. *C. glutamicum* CP cells from the stationary phase of the fed-batch cultures were collected to study the effects of ultrasonic treatment on enzyme activity, as shown in Figure 7. The relative activity of AHAS was not significantly different between the ultrasonically treated culture and the control. However, the relative activity of IPMS and IPMD under ultrasonication significantly increased by 8.9% and 18.2% compared to the control, respectively. Cavitation generated by ultrasound can lead to conformational changes of enzymes, which can improve the contact between the enzyme and substrate, which in turn can increase the enzyme activity [16,20,39,40]. Moreover, ultrasound treatment can improve the solubility and dispersion of recalcitrant substrates, greatly improving their degradation and utilization. For example, when *L. acidophilus* BCRC 10695 was treated with ultrasound (20 kHz, amplitude at 20%) during the stationary phase, the β-glucosidase activity was enhanced to 3.91 U/mL, which was 82% times higher than without ultrasound treatment [41]. Thus, the improvement of enzyme activity observed in this study was in agreement with the literature, and can explain at least a part of the improved L-leucine production under ultrasound treatment.

## Conclusions

In this study, four factors of ultrasound treatment, including the power density, frequency, interval, and duration, were optimized to improve the biomass and the production of L-leucine by *C. glutamicum* CP in fed-batch culture. Moreover, the yield and productivity of L-leucine were significantly improved. The enhancement of L-leucine production was attributed to intense micro-mixing induced by sonication, which has several possible effects on cell morphology, cell membrane permeability, and enzyme activity. This is a successful case of process intensification using ultrasound, and is the first report of ultrasound-assisted L-leucine production. Furthermore, we consider that the ultrasound effect of L-leucine producing strain *C. glutamicum* CP in fed-batch culture should be further studied by various omics.

## Supplementary Material

Supplemental Material

## Highlights

The optimal combination of ultrasonic parameters was determined by RMS.

The yield and productivity of L-leucine were improved with ultrasound treatment.

The permeability of cell membrane and the activities of key enzymes were significantly improved by ultrasound.
