# Supplementary material for: Effect of low-level ultrasound treatment on the production of L-leucine by Corynebacterium glutamicum in fed-batch culture
Source: Bioengineered. 2021 Mar 29;12(1):1078–90. doi: 10.1080/21655979.2021.1906028 (PMC8806274; doi:10.1080/21655979.2021.1906028)
Supplement: Supplemental Material [file KBIE_A_1906028_SM0471.docx]

**Additional file for:**

**Effect of low-level ultrasound treatment on the production of L-leucine by *Corynebacterium glutamicum* in fed-batch culture**

Yufu Zhang^1,2^, Zhichao Chen^1,2^, Pengjie Sun^1,2^, Qingyang Xu^1,2^*, Ning Chen^1,2^*

1. National and Local United Engineering Lab of Metabolic Control Fermentation Technology, Tianjin University of Science & Technology, Tianjin 300457, PR China

2. College of Biotechnology, Tianjin University of Science & Technology, Tianjin 300457, PR China

*Corresponding author:

Dr. Qingyang Xu, College of Biotechnology, Tianjin University of Science & Technology, No. 29, 13 Main Street, Tianjin Economic and Technological Development Area, Tianjin 300457, PR China; Tel: 86-22-60601251; Fax: +86-022-60601251; E-mail: xuqingyang@tust.edu.cn

Dr. Ning Chen, College of Biotechnology, Tianjin University of Science & Technology, No. 29, 13 Main Street, Tianjin Economic and Technological Development Area, Tianjin 300457, PR China; Tel: 86-22-60601251; Fax: +86-022-60601251; E-mail: [chenning608@126.com](mailto:chenning608@126.com)

E-mail addresses of other authors:

Y. Zhang: yfz362@sina.com;

Z. Chen: czc101x@163.com;

P. Sun: 1559134227@qq.com;

**Fig. S1** Schematic of ultrasonic device and fermentation system

**Table S1**: Actual and coded level of factors tested with Box–Behnken design

| Code | Factor | Unit | Coded levels | | |
| --- | --- | --- | --- | --- | --- |
|  |  |  | -1 | 0 | 1 |
| A | Power density | W/L | 60 | 100 | 140 |
| B | Frequency | kHz | 20 | 25 | 30 |
| C | Interval | min | 20 | 30 | 40 |
| D | Duration | s | 30 | 40 | 50 |

**Table S2**: Box–Behnken design (BBD) experimental design with independent variables and experimental data for the responses.

| Run | Power density (W/L)  A | Frequency (kHz)  B | Interval (min)  C | Duration (s)  D | L-leucine titer ( g/L)  Y |
| --- | --- | --- | --- | --- | --- |
| 1 | 60.00 | 25.00 | 30.00 | 30.00 | 43.35 |
| 2 | 100.00 | 25.00 | 40.00 | 30.00 | 47.20 |
| 3 | 100.00 | 30.00 | 20.00 | 40.00 | 39.04 |
| 4 | 60.00 | 30.00 | 30.00 | 40.00 | 43.07 |
| 5 | 100.00 | 30.00 | 40.00 | 40.00 | 39.11 |
| 6 | 100.00 | 20.00 | 30.00 | 30.00 | 31.14 |
| 7 | 60.00 | 25.00 | 30.00 | 50.00 | 42.59 |
| 8 | 100.00 | 25.00 | 20.00 | 30.00 | 40.61 |
| 9 | 140.00 | 25.00 | 40.00 | 40.00 | 41.87 |
| 10 | 60.00 | 25.00 | 20.00 | 40.00 | 45.94 |
| 11 | 100.00 | 20.00 | 30.00 | 50.00 | 29.84 |
| 12 | 140.00 | 20.00 | 30.00 | 40.00 | 31.93 |
| 13 | 100.00 | 25.00 | 30.00 | 40.00 | 53.10 |
| 14 | 140.00 | 25.00 | 20.00 | 40.00 | 26.63 |
| 15 | 100.00 | 30.00 | 30.00 | 30.00 | 43.86 |
| 16 | 100.00 | 25.00 | 40.00 | 50.00 | 38.87 |
| 17 | 100.00 | 25.00 | 20.00 | 50.00 | 35.93 |
| 18 | 100.00 | 25.00 | 30.00 | 40.00 | 52.96 |
| 19 | 100.00 | 20.00 | 40.00 | 40.00 | 39.48 |
| 20 | 100.00 | 30.00 | 30.00 | 50.00 | 35.28 |
| 21 | 140.00 | 30.00 | 30.00 | 40.00 | 35.76 |
| 22 | 100.00 | 20.00 | 20.00 | 40.00 | 25.22 |
| 23 | 100.00 | 25.00 | 30.00 | 40.00 | 52.31 |
| 24 | 140.00 | 25.00 | 30.00 | 50.00 | 34.18 |
| 25 | 60.00 | 25.00 | 40.00 | 40.00 | 40.73 |
| 26 | 100.00 | 25.00 | 30.00 | 40.00 | 52.14 |
| 27 | 100.00 | 25.00 | 30.00 | 40.00 | 51.63 |
| 28 | 60.00 | 20.00 | 30.00 | 40.00 | 29.29 |
| 29 | 140.00 | 25.00 | 30.00 | 30.00 | 38.05 |

**Table S3**: Variance analysis of the parameters of L-leucine titers in the regression equation.

| Source of variation | Sum of Squares | Degree of freedom | Mean square | F-Value | p-value Prob > F |
| --- | --- | --- | --- | --- | --- |
| Model | 1719.67 | 14 | 122.83 | 38.49 | < 0.0001 |
| A | 111.52 | 1 | 111.52 | 34.94 | < 0.0001 |
| B | 201.74 | 1 | 201.74 | 63.21 | < 0.0001 |
| C | 95.68 | 1 | 95.68 | 29.98 | < 0.0001 |
| D | 63.09 | 1 | 63.09 | 19.77 | 0.0006 |
| AB | 24.75 | 1 | 24.75 | 7.75 | 0.0146 |
| AC | 104.55 | 1 | 104.55 | 32.76 | < 0.0001 |
| AD | 2.42 | 1 | 2.42 | 0.76 | 0.3985 |
| BC | 50.34 | 1 | 50.34 | 15.77 | 0.0014 |
| BD | 13.26 | 1 | 13.26 | 4.15 | 0.0609 |
| CD | 3.33 | 1 | 3.33 | 1.04 | 0.3245 |
| A2 | 317.67 | 1 | 317.67 | 99.53 | < 0.0001 |
| B2 | 755.86 | 1 | 755.86 | 236.82 | < 0.0001 |
| C2 | 240.83 | 1 | 240.83 | 75.46 | < 0.0001 |
| D2 | 238.24 | 1 | 238.24 | 74.64 | < 0.0001 |
| Residual Error | 44.68 | 14 | 3.19 | — | — |
| Lack of Fit | 43.21 | 10 | 4.32 | 11.76 | 0.0149 |
| Pure Error | 1.47 | 4 | 0.37 | — | — |
| Total | 1764.36 | 28 | — | — | — |

A is the ultrasound power density, B is the ultrasound frequency, C is the ultrasonication interval, and D is the duration of ultrasonication.

R^2^ = 0.9747, Adj R^2^ = 0.9493, CV% = 4.46.
